# Disability trajectories in the last years of life: a Swedish longitudinal multi-cohort study

**DOI:** 10.1093/gerona/glag226

**Published:** 2026-09-10

**Authors:** Saila Kyrönlahti, Pauliina Halonen, Debora Rizzuto, Anna-Karin Welmer, Linda B Hassing, Deborah Finkel, Stefan Fors, Johan Sanmartin Berglund, Linda Enroth

**Affiliations:** Faculty of Social Sciences, Unit of Health Sciences and Gerontology Research Center, Tampere University, Tampere, Finland; Finnish Institute for Health and Welfare, Department of Public Health, Helsinki, Finland; Faculty of Social Sciences, Unit of Health Sciences and Gerontology Research Center, Tampere University, Tampere, Finland; Finnish Institute for Health and Welfare, Department of Healthcare and Social Welfare, Services Unit, Helsinki, Finland; Aging Research Center, Department of Neurobiology, Care Sciences and Society, Karolinska Institutet and Stockholm University, Stockholm, Sweden; Aging Research Center, Department of Neurobiology, Care Sciences and Society, Karolinska Institutet and Stockholm University, Stockholm, Sweden; Division of Physiotherapy, Department of Neurobiology, Care Sciences and Society, Karolinska Institutet, Stockholm, Sweden; Women’s Health and Allied Health Professionals Theme, Medical Unit, Medical Psychology, Karolinska University Hospital, Stockholm, Sweden; Department of Psychology, University of Gothenburg, Gothenburg, Sweden; Centre for Ageing and Health, University of Gothenburg, Gothenburg, Sweden; Institute for Gerontology, School of Health and Welfare, Jönköping University, Jönköping, Sweden; Center for Economic and Social Research & Department of Psychology, University of Southern California, Los Angeles, California, United States; Aging Research Center, Department of Neurobiology, Care Sciences and Society, Karolinska Institutet and Stockholm University, Stockholm, Sweden; Centre for Epidemiology and Community Medicine, Region Stockholm, Stockholm, Sweden; Department of Health, Blekinge Institute of Technology, Karlskrona, Sweden; Faculty of Social Sciences, Unit of Health Sciences and Gerontology Research Center, Tampere University, Tampere, Finland

**Keywords:** Activities of daily living, Aging, Group-based trajectory modeling, Time-to-death, Cognitive impairment

## Abstract

**Background:**

The patterns and determinants of late-life functional decline are not fully understood. We examined disability trajectories in relation to proximity to death among decedents aged 70 years and older and investigated their associations with sex, age at death, education, cognitive impairment, and chronic conditions.

**Methods:**

Harmonized data from six Swedish longitudinal aging studies (1997-2016) within the National E-infrastructure for Aging Research (NEAR) were used. Participants who died at age 70 years or older and had data on activities of daily living (ADL) from at least two assessments were included (*N* = 2294, 62% women, mean age at death 89.8 years [*SD* 6.5]). ADL limitations (0-5 scale) were analyzed across four time intervals before death (0-2, >2-4, >4-6, and 6-12 years). Group-based trajectory modelling identified disability trajectories, multinomial logistic regression examined demographic associations, and generalized estimating equations assessed associations of cognitive impairment and chronic conditions with trajectory membership.

**Results:**

Three trajectories were identified: *constantly low* (53%), *low-sharply increasing* (36%), and *high-increasing* (11%). Women, those with lower education, and individuals dying at older ages were more likely to follow increasing or persistently high disability trajectories. *A high-increasing* trajectory was associated with a higher likelihood of cognitive impairment.

**Conclusions:**

For most, ADL limitations remained low until the final two years of life, though distinct trajectories with steady or sharp increases also emerged. Identifying demographic and health-related factors associated with functional decline may inform targeted interventions and help anticipate care needs.

## Introduction

Functional limitations in activities of daily living (ADLs)^1^ are a key marker of health and autonomy in old age,^2^ reflecting increased care needs and contributing substantially to the burden on families and healthcare systems. Functional decline is strongly associated with proximity to death, as most older adults experience a period of disability in their final years.^3^^,^^4^ However, the duration, progression, and severity of this decline vary widely.^5–9^ Some individuals experience a slow deterioration extending over many years, and others a rapid, late-life loss of function.

Advances in medical care have enabled more people, including those with significant frailty, to survive into very old age, potentially extending periods of poor health and disability. This *expansion of morbidity*^10^ would increase demand for healthcare and long-term care. Conversely, improvements in disease prevention, treatment, and living conditions may lead to a *compression of morbidity,*^11^ delaying functional decline and shortening the time spent with functional limitations.^12^ Understanding variation in functional decline in the years preceding death and identifying factors associated with distinct trajectories is critical for anticipating care needs, targeting preventive strategies, and planning services for aging populations.

Older age is consistently associated with greater severity of functional limitations, whereas female sex and lower educational attainment are linked to both greater severity and longer duration of disability.^13–16^ These associations are, however, shaped by selective survival. Women and individuals with higher education tend to live longer, spending more years at risk of disability, which likely extends the duration of late-life limitations.^13^ Functional decline in old age can be understood within a disablement process framework, in which chronic conditions act as key proximal determinants leading to impairments and eventually functional limitations and disability.^17^ In addition to basic biological factors, age and sex, life-course factors, such as educational attainment, and cohort-related differences in living conditions and health care may influence both the risk of disease and the progression of functional decline.

Within this framework, chronic conditions differ in their contribution to late-life disability. Cognitive impairment, particularly dementia, is a leading cause of prolonged ADL limitations and disability.^5^^,^^9^ Other chronic conditions, including cardiovascular disease, stroke, and cancer, may also contribute to disability through different pathways, such as physical impairments, reduced physiological reserve, symptom burden, or treatment-related effects.^18^^,^^19^ Consequently, these conditions may be associated with different patterns and rates of functional decline in late life. However, their roles in the progression of disability trajectories during the last years of life remain unclear.

To address bias arising from differential survival, a mortality follow-back approach, in which functional trajectories are derived retrospectively from the time of death, can provide clearer evidence on determinants of late-life disability. Despite extensive research on functional decline, few studies have addressed disability trajectories in relation to impending death. Most rely on calendar age or disease-specific trajectories or have follow-up periods too short to capture long-term patterns.^3–6^^,^^20^^,^^21^ Studying the oldest old presents additional challenges due to high attrition rates, as those most vulnerable to functional decline and mortality are also the most likely to drop out. Consequently, single-cohort studies often lack sufficient statistical power to examine heterogeneous trajectories in the final years of life.

To address prior limitations, we used harmonized data from multiple Swedish aging studies and a person-centered analytical approach within a mortality follow-back design. Our first aim was to identify disability trajectories in the years preceding death among individuals who died at age 70 or older. Second, we examined whether these trajectories differed by age at death, sex, and education and assessed the role of cognitive impairment and chronic conditions in shaping late-life disability trajectories.

## Methods

### Study design and population

This study is based on harmonized data from the Swedish National E-infrastructure for Aging Research study (NEAR, https://www.near-aging.se/). NEAR coordinates the existing databases from major population-based longitudinal studies on aging and health in Sweden, providing harmonized variables and documentation generated by a dedicated team of data analysts, statisticians, and epidemiologists. The six cohort studies included in this study are the Betula Project (Betula), Aging in Women and Men: A Longitudinal Study of Gender Differences in Health Behavior and Health among Elderly (Gender), Origin of Variances in the Oldest-Old: Octogenarian Twins (Octo-Twin), the Swedish National Study of Age and Care—Blekinge (SNAC-B), the Swedish National Study of Age and Care—Kungsholmen (SNAC-K), and the Swedish Panel Study of Living Conditions of the Oldest Old (SWEOLD). (Detailed description in Table S1). The contributing studies used either self-administered questionnaires or interviewer-administered assessments. In three cohorts (SNAC-B, SNAC-K, and SWEOLD), proxy respondents could be used when needed, allowing participation of individuals who might otherwise have been unable to provide reliable self-reported information. In the SNAC studies, a spouse or partner, adult child, other relative, or professional caregiver could serve as a proxy respondent.^22^ The informant was identified by the participant, and an informant interview was conducted when additional information was needed. In SWEOLD, proxy interviews were primarily conducted when participants were unable to respond because of dementia or frailty, and proxies included close relatives, trustees, and healthcare personnel.^23^

Together, these studies comprised a total of 4801 individuals who had died at the age of 70 years or older by the time of data harmonization. Mortality data were obtained from the National Cause of Death Registry. Time-to-death was used as the study’s time scale; the decedents were followed retrospectively for up to 12 years prior to their year of death. We included those with information on ADL functioning from at least two assessments during the follow-up. Individuals who participated in only one study wave, as well as those with ADL data available at fewer than two assessments because of missing ADL information, were excluded. The final analytic sample consisted of 2294 individuals drawn from the six studies (Figure S1).

Attrition analyses were conducted to compare participants included in the analytical sample with those excluded because of insufficient follow-up data. Excluded participants were older at death, less educated, and more likely to belong to earlier birth cohorts than those included in the analyses (Table S2).

The study was conducted in accordance with the Declaration of Helsinki and approved by the Swedish Ethical Review Authority (Drn: 2023-01193-01). Each original study obtained informed consent from participants according to regulations valid at the time of data collection.

### Assessment of disability

Disability was assessed with the number of limitations in basic ADLs, a validated measure of disability, with a higher number of limitations indicating greater dependence.^21^ ADL limitations were evaluated across four time intervals: 0-2 years, >2-4 years, >4-6 years, and >6-12 years before death. If multiple responses were available within a given interval, the response closest to death was used.

ADL limitations were based on participants’ self-reported ability to perform five daily self-care activities from the validated Katz Activities of Daily Living questionnaire^1^: dressing, bathing/showering, eating, getting in/out of bed, and toileting. The continence item included in the original Katz Index was not available across all participating cohorts and was therefore excluded to ensure comparability of the harmonized ADL measure. For each item, participants were categorized as having no limitations (no difficulty) or a functional limitation (any difficulty or inability). The number of ADL limitations at each time point was calculated as the total number of items with functional limitations.

On average, respondents provided data at 2.3 time points (*SD* 0.5) across the four intervals.

### Independent variables

Demographic factors included age at death, sex (male/female), and education. Education was categorized based on the International Standard Classification of Education (ISCED) version 2011, which provides a standardized measure of educational attainment for comparative analyses across populations and birth cohorts. The three categories were low (0-2 = primary and lower secondary education), intermediate (3-5 = upper secondary education, vocational education, and academic tertiary education programs below the level of bachelor’s program/short-cycle tertiary education), and high (6-8 = tertiary education, bachelor’s, master’s, and doctoral level).

Information about diseases was collected at each assessment and included cardiovascular disease (CVD), stroke, and cancer. Cognition was measured with the Mini-Mental State Examination (MMSE). MMSE scores were harmonized into a binary indicator of possible cognitive impairment. In most cohorts, a cutoff of <24 points was used, whereas SWEOLD employed an abbreviated 18-point MMSE and a cohort-specific cutoff of <8 points.^24^

### Statistical analysis

An unconditional group-based trajectory modeling (GBTM) was used to investigate trajectories of disability in relation to proximity of death and identify subgroups with similar disability patterns. GBTM, a person-centered finite mixture modeling approach, detects latent subgroups within a dataset that follow distinct patterns of change over time for a given variable.^25^^,^^26^

The best-fitting model was identified iteratively, as described by Nagin (2005, pp. 61-77)^26^: first, the optimal number of classes was determined, and second, the most suitable polynomial forms for each class were specified.

Trajectory analyses were conducted with the Stata *traj* package(StataCorp LLC, College Station, TX).^27^ A zero-inflated Poisson distribution was specified to account for repeated count data (number of ADL limitations) with an excess of zeros.^27^ Models with up to five latent classes were tested, starting with quadratic-shaped trajectories. The polynomial order for each class was subsequently adjusted to identify the most parsimonious model with meaningful trajectories.

The best-fitting model was selected based on several statistical fit indices (Table S3), the size of the latent classes (minimum 5% of the sample), and interpretability. Fit indices included the Bayesian Information Criterion and Akaike Information Criterion, where lower values indicated better fit. Additional criteria included posterior probabilities of class membership >0.70 and substantive interpretation of the derived trajectories. Entropy, reflecting the degree of separation between trajectory groups, and the certainty of individual classification, was considered alongside other model fit and interpretability criteria. Higher entropy values indicate clearer classification.^28^

As a sensitivity analysis, trajectory modeling was repeated among participants with ADL information available at more than two time points to assess whether the identified trajectory structure was influenced by participants contributing only two observations.

Participants were assigned to a trajectory group for which they had the highest posterior probability of membership. Subsequent post-estimation analyses were then conducted. Demographic characteristics were summarized as means and standard deviations (*SDs*) for continuous variables and as proportions for categorical variables, stratified by trajectory membership. *p*-values for between-trajectory differences were derived from chi-square tests for categorical variables and one-way ANOVA for age at death. Trajectory membership was then used as the dependent variable in multinomial regression models to investigate univariate associations with demographic factors, presented as average marginal effects (AMEs) with their 95% confidence intervals (CIs). AMEs quantify the average change in the probability of trajectory membership associated with each independent variable. To account for the skewed distribution and to facilitate interpretation, age at death was standardized prior to modeling. Estimates are presented per one *SD* increase (*SD* = 6.50 years).

Generalized estimating equation (GEE) modeling was used to analyze the prevalence of cognitive impairment and chronic conditions across trajectories over time. A binomial distribution with a logit link function was applied, and an independent working correlation structure accounted for intra-individual correlations in repeated measures.

Attrition analyses were conducted to evaluate potential biases due to sample attrition. Differences between participants included in the analysis and those excluded (individuals with ADL data available only at one assessment) were assessed using chi-square tests and ANOVA.

## Results

The demographic characteristics of the respondents for the whole study sample and by disability trajectories are shown in Table 1. The average age of death was 89.8 years (*SD* 6.5), and 61% of the participants were women. Slightly more than half of the participants had a low level of education, and the majority (88%) were born before 1930. Participant characteristics across the four time-to-death intervals and the availability of longitudinal ADL data are presented in Table S5.

**Table 1:** Demographic characteristics of the study population by disability trajectories.

	All (*n* = 2294)	Constantly low (*n* = 1336)	Low-sharply increasing (*n* = 743)	High-increasing (*n* = 215)	*p* for difference^a^
Women, %	61.2	55.2	66.9	78.6	<.001
Age at death, years, mean (*SD*)	89.8 (6.5)	88.2 (6.6)	91.7 (5.5)	92.6 (6.5)	<.001
**Birth cohort**					<.001
1900-1918	41.7	32.6	53.0	58.6	
1919-1929	46.1	50.1	42.3	34.4	
1930-1944	12.3	17.3	4.7	7.0	
**Education level, %**					<.001
low	56.6	52.1	62.9	63.7	
intermediate	31.8	34.4	28.3	27.9	
high	11.5	13.5	8.8	8.5	
**Study, %**					<.001
Betula	12.6	15.3	8.9	8.4	
Gender	3.0	1.5	6.2	1.4	
Octo-Twin	6.7	5.2	8.5	9.8	
Snac_B	25.5	25.8	24.8	25.6	
Snac_K	38.2	38.0	37.0	43.7	
SWEOLD	14.0	14.1	14.7	11.6	

Calculated among participants with non-missing data. Missing data included: education *n* = 66.

a Derived from chi-square tests for categorical variables and one-way ANOVA for age at death.

### Disability trajectories

Model fit indices (Table S3) indicated that the best-fitting solution was a three-trajectory model, comprising one trajectory with a linear slope and two with quadratic shapes (Figure 1). The average posterior probabilities were high (range 0.84-0.88), with an entropy value of 0.68 demonstrating moderate class separation.^29^ The smallest trajectory accounted for 11% of the sample, meeting the recommended threshold.^25^ Parameter estimates for the selected model are presented in Table S4.

**Figure 1:**
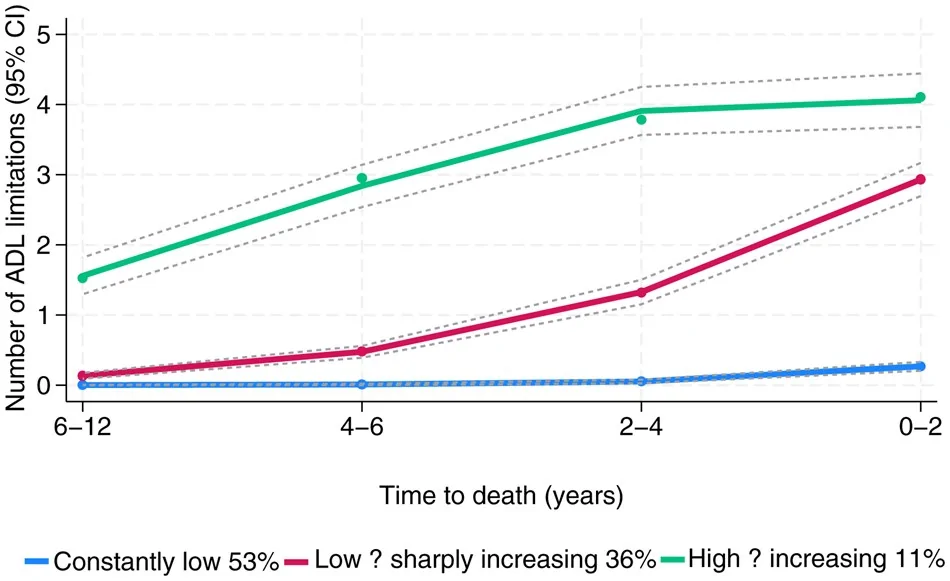
Disability trajectories in relation to impending death. ADL = activities of daily living. Trajectory 1: constantly low (*n* = 1336); trajectory 2: low-sharply increasing (*n* = 743); trajectory 3: high-increasing (*n* = 215).

The most common trajectory, representing 53% of the sample, was characterized by a consistently low number of ADL limitations throughout the follow-up period. The estimated mean number of ADL limitations remained close to zero from 6-12 years to 2-4 years before death (0.0-0.1 limitations) and increased modestly during the final two years of life to 0.3 limitations. The second-largest trajectory, encompassing 36% of participants, showed a low but steadily increasing number of ADL limitations, rising from 0.1 limitations 6-12 years before death to 0.5 limitations 4-6 years before death and 1.3 limitations 2-4 years before death, followed by a sharp increase to 2.9 limitations during the final 2 years of life. The smallest trajectory, comprising 11% of the sample, was marked by ADL limitations already present 6-12 years before death (1.6 limitations), increasing to 2.8, 3.9, and 4.1 limitations as death approached.

Seventy percent of participants contributed ADL information at only two observation intervals. Participants contributing ADL information at only two time points differed somewhat from those with more extensive follow-up, being younger at death and having lower educational attainment (Table S6). Nevertheless, in sensitivity analyses restricted to participants with ADL information available at more than two time points (*n* = 699), the three-trajectory solution remained optimal (Table S7), and the resulting trajectory shapes were highly similar to those observed in the full analytical sample (Figure S2).

### Associations of sociodemographic factors with disability trajectories

The identified disability trajectories differed substantially in demographic characteristics (Table 2). Higher age at death, low educational attainment, and being a woman were associated with an increased likelihood of membership in *low-sharply increasing* and *high-increasing* disability trajectories. (Table 2).

**Table 2:** Associations of sociodemographic factors with disability trajectories: average marginal effects (AMEs) with their 95% confidence intervals (CIs) from univariate multinomial logistic regression models.

	Constantly low	Low-sharply increasing	High-increasing
	AME (95% CI)	AME (95% CI)	AME (95% CI)
Age at death, z-score^a^	−0.14 (−0.16 to −0.12)	0.10 (0.08 to 0.11)	0.04 (0.03 to 0.06)
Sex			
female	ref.	ref.	ref.
male	0.15 (0.11 to 0.19)	−0.08 (−0.12 to −0.04)	−0.07 (−0.09 to −0.05)
Education level			
Low	−0.15 (−0.21 to −0.08)	0.11 (0.05 to 0.17)	0.04 (0.01 to 0.07)
intermediate	−0.05 (−0.12 to 0.01)	0.04 (−0.02 to 0.10)	0.01 (−0.02 to 0.05)
High	ref.	ref.	ref.

a Standardized to the sample mean and scaled by standard deviation.

### Prevalence of cognitive impairment and chronic conditions across disability trajectories

Figure 2 shows the prevalence of cognitive impairment and major diseases across disability trajectories in the years before death. The prevalence of cognitive impairment increased across all trajectories as death approached (Panel A) and was consistently higher in *low-sharply increasing* and *high-increasing* trajectories than in the *constantly low* trajectory. Between 2 and 4 years before death, prevalence was also higher in the *high-increasing* than in *low-sharply increasing* trajectory [0.80 (95% CI 0.72-0.88) vs 0.46 (95% CI 0.42-0.51)], but this difference disappeared during the final 0-2 years as prevalence rose steeply in the *low-sharply increasing* trajectory [0.81 (95% CI 0.73-0.89) vs 0.70 (95% CI 0.65-0.74)].

**Figure 2:**
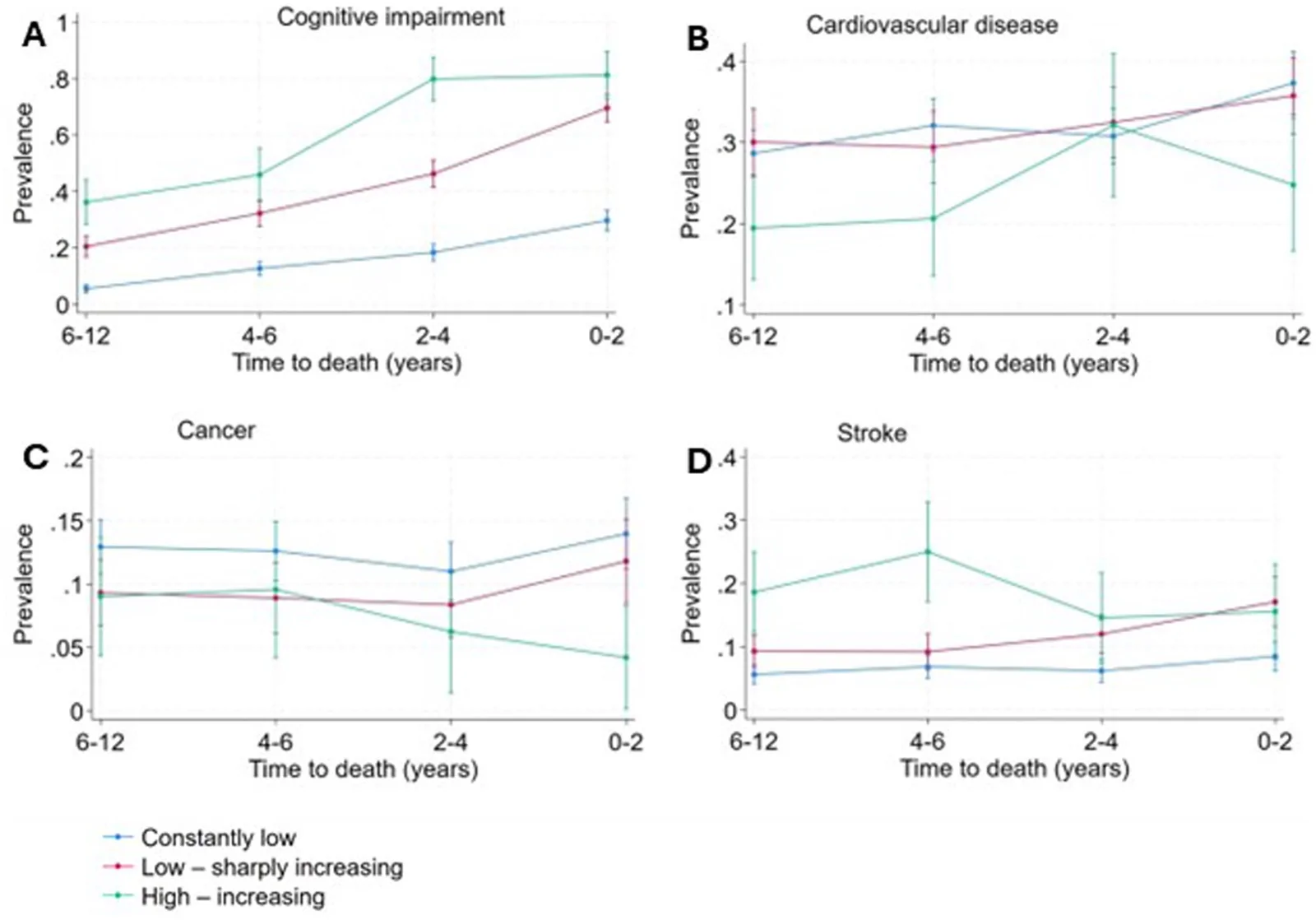
Prevalence of cognitive decline (A) and, cardiovascular disease, cancer, and stroke (B-D) across disability trajectories in the years before death.

For CVD and cancer (Panels B and C), prevalence estimates showed some between-trajectory variation, but the differences were not statistically significant. Both conditions slightly increased as death approached, except in the *high-increasing* trajectory, where probabilities declined during the last two years of life. In contrast, stroke (Panel D) was more common in the *high-increasing* trajectory 12-2 years before death, whereas in the *low-sharply increasing* trajectory prevalence rose steadily and overtook the other groups in the final 0-2 years, though again differences were not statistically significant.

## Discussion

In this large, multicohort Swedish study, we identified three distinct disability trajectories in the twelve years preceding death. Most individuals who died at age 70 years or older remained free of disability until the final two years of life. However, around one in ten experienced limitations in multiple ADLs already 6-12 years before death, and for more than one third, ADL limitations increased steadily during follow-up, indicating a prolonged need for assistance in daily life. Our findings show significant sociodemographic disparities: women and individuals with lower levels of education were more likely to experience longer and more severe periods of disability at the end of life. Older age at death also predicted a longer duration of functional decline. Cognitive impairment emerged as the most important factor associated with the need for assistance.

The use of a data-driven approach, in which the identified disability trajectories are derived under specific modeling assumptions, limits direct comparisons with previous studies, as differences in timing of functional assessments relative to death, frequency of follow-up measurements, and the metrics used to define functioning may all influence the number and shape of the trajectories. GBTM, however, is valuable for capturing heterogeneity in the development of disability that might remain hidden in population-averaged analyses. In the present study, we focused on basic ADLs, as these represent essential self-care functions and capture limitations that reflect the need for help and dependency in late life. In addition, only a few previous studies have tracked changes in functional ability until death, and those that did typically focused on the period in close proximity to death, rather than over a longer timespan as in our study.^3^^,^^5^^,^^6^

Despite these methodological differences, our findings are consistent with previous research demonstrating considerable heterogeneity in the development of functional limitations and disability in late life.^4–6^^,^^9^^,^^30^ For example, Edjolo et al. (2020)^9^ identified five distinct trajectories over the last two decades of life in the general population, ranging from persistent or accelerated disability to no disability. In line with our results, most individuals maintained functional ability until the final two years of life, when mild dependency emerged, whereas a smaller but notable proportion lived several years with a high level of limitations and required assistance with activities of daily living for at least two to four years before death.

### Sociodemographic factors associated with disability trajectories

Extensive research demonstrates that women experience both greater severity and longer duration of disability than men. This disparity is largely attributable to earlier onset, lower recovery rates, and longer survival with functional limitations.^31^ Educational attainment is also a well-established determinant of disability and mortality.^32^ Due to uneven distribution of material and social resources and differences in health behaviors, lower education has consistently been associated with earlier onset and higher prevalence of disability.^33^

Our findings support the disablement process framework,^17^ in which sex and educational attainment act as distal determinants that shape the development and progression of disability through accumulated differences in health, resources, and resilience across the life course. Consistent with this framework, women and individuals with lower educational attainment were more likely to follow progressive and persistently severe disability trajectories. These findings are also in line with previous studies that have examined disability trajectories retrospectively from the time of death, thereby minimizing survival bias. Both long-term^9^ and short-term follow-up studies^5^ have similarly indicated that women and individuals with lower education are more likely to experience prolonged and severe disability before death.

Our study further demonstrated that both older age at death and proximity to death were associated with an increasing number of ADL limitations. In the last interval, 0-2 years before death, nearly half of participants had limitations in three or more ADLs. As these activities represent basic functions essential for independent living, our findings indicate substantial personal care needs in the last years of life. These needs are expected to grow as the number of people living their last years of life in very old age continues to increase.

### Chronic conditions and cognitive impairment in relation to disability trajectories

Within the disablement process framework, chronic conditions and cognitive impairment are considered proximal determinants of disability, which directly contribute to functional decline in late life. Previous research has shown a strong association between cognitive impairment, dementia, and late-life disability.^5^^,^^9^^,^^30^ Declining cognition can limit the ability to plan, initiate, and perform everyday activities,^34^ thereby increasing the need for assistance and contributing to long-term care dependency.^35^ In our study, cognitive impairment was most common among individuals following the *high-increasing* disability trajectory, characterized by ADL limitations already present 6-12 years before death. In line with previous research, this finding suggests that cognitive decline may contribute particularly to an earlier onset and longer duration of disability before death. Given the projected global rise in dementia,^36^ this association is increasingly important for predicting future care needs in aging populations.

Cognition and vascular health are closely linked in late-life disability. Stroke, for example, has a bidirectional relationship with cognitive decline: cerebrovascular damage may impair cognition, while cognitive impairment can increase vulnerability to stroke-related disability.^18^ Stroke was more frequent in the *high-increasing* trajectory up to two years before death, while in the *low sharply increasing* trajectory its prevalence rose later and exceeded that of the other groups near death. These results may reflect the contribution of stroke to both the early onset of disability and the rapid functional decline observed in the last years of life, as stroke often results in sudden neurological impairments affecting mobility, self-care, and other ADLs.

Cardiovascular diseases are associated with functional decline through multiple pathways, including reduced physical capacity, impaired mobility, and increased burden of comorbidity. Although CVD prevalence did not differ significantly between disability trajectories in our study, CVDs are known to contribute substantially to disability burden, particularly among men,^37^,and are more likely to lead to death at younger ages.^38^ Those who survive to older ages may delay the onset of CVDs or the onset of disability, and especially women live with these diseases longer than men.^39^^,^^40^ The improved medical treatment and preventive actions to lower the burden of CVDs have enabled people to live with these conditions longer and most likely also lowered the disability burden of CVDs.^38^^,^^40^

Similarly, due to population aging and advances in cancer treatment, more people are living longer with cancer. Functional decline is a common consequence of cancer, often resulting from symptom burden or muscle weakness. Unlike stroke, which often results in immediate neurological impairments affecting mobility and self-care, cancer comprises a heterogeneous group of diseases with highly variable effects on functioning depending on cancer type, stage, treatment, and proximity to death. Many individuals may maintain independence in basic ADLs for prolonged periods despite a cancer diagnosis, particularly when disability develops only in the final phase of illness.

In our study, cancer diagnosis appeared somewhat more common among individuals belonging to a *constantly low disability* trajectory, characterized by the lowest age at death and a higher proportion of men. This observation was not statistically significant and should therefore be interpreted cautiously. Nevertheless, previous studies have shown that disability trajectories preceding cancer-related death can vary substantially. Previous research on disability trajectories prior to death among individuals dying of cancer suggests either a steady increase beginning 10 years prior to death^8^ or a catastrophic, accelerated increase during the last months.^5^ A review examining disability trajectories in advanced cancer also found progressive or sudden increases prior to death but, similar to our findings, reported stable trajectories as well.^19^

### Strengths and limitations

This study has several notable strengths. First, the use of harmonized data from six well-established longitudinal Swedish aging studies provided an exceptionally rich and diverse dataset, enabling robust modeling of disability trajectories among individuals in their last years of life. The multicohort design also mitigated the effects of attrition that commonly affects studies of the oldest old. Second, the application of a person-centered GBTM approach allowed us to capture heterogeneity in disability patterns that would otherwise be masked in population-averaged analyses, thereby addressing limitations of previous research often constrained by shorter follow-up periods or less refined methodologies.

Nonetheless, limitations should also be acknowledged. Although harmonization improved comparability, differences in study design, sampling, and measurement protocols may have introduced residual heterogeneity that could not fully be eliminated. Data collection procedures also varied between cohorts, including the use of proxy respondents in some studies, which may have influenced measurement comparability. In three cohorts, proxy respondents could be used when participants were unable to provide information themselves. While the use of proxies enabled the inclusion of individuals with severe cognitive or functional impairment who might otherwise have been excluded, proxy assessments may differ from participants’ own reports and may therefore have introduced additional measurement variability.

The two-step modeling approach used in this study, first identifying trajectories and then assigning individuals to their most probable trajectory, may have introduced some classification bias, potentially attenuating the observed associations. Although the average posterior probabilities indicated acceptable classification quality (0.84-0.88), the entropy value suggested moderate classification accuracy and some uncertainty in individual group assignments.

Most participants contributed ADL information at only two time points. However, sensitivity analyses restricted to participants with more extensive longitudinal follow-up yielded highly similar trajectory patterns (Figure S2), supporting the robustness of the identified trajectories. Finally, limitations also concern the measurement of ADL, as it is a coarse indicator of functioning, reflecting both physical and cognitive abilities as well as environmental influences. Information on behavioral and environmental factors affecting disability trajectories was lacking. In addition, ADL limitations were primarily self-reported, and some reporting bias may have occurred among individuals with cognitive impairment. Nevertheless, self-reported ADL measures are widely used and well established in epidemiological studies of aging and disability.

## Conclusions

This study demonstrates considerable heterogeneity in disability trajectories beginning as early as 6-12 years prior to death and confirms that self-care needs intensify markedly as death approaches. Women, individuals with low educational attainment or cognitive impairment, and those who died at more advanced ages were disproportionately represented in trajectories characterized by initially high and sharply increasing disability. Rising life expectancy has shifted the final years of life to progressively older ages, where cognitive impairment becomes more prevalent, and women outlive men. These trends suggest that with continued increases in life expectancy, care needs in old age will grow significantly.

## Supplementary Material

glag226_Supplementary_Data

## Data Availability

Data are from the Betula Project (Betula), Aging in Women and Men: A Longitudinal Study of Gender Differences in Health Behavior and Health among Elderly (Gender), Origin of Variances in the Oldest-Old: Octogenarian Twins (Octo-Twin), the Swedish National Study of Age and Care—Blekinge (SNAC-B), the Swedish National Study of Age and Care—Kungsholmen (SNAC-K), and the Swedish Panel Study of Living Conditions of the Oldest Old (SWEOLD), and were obtained from the National E-infrastructure for Aging Research (NEAR). The data are not publicly available due to ethical restrictions but can be accessed through the Swedish National E-infrastructure for Aging Research (NEAR) upon approved application.
