# Supplementary material for: Disability trajectories in the last years of life: a Swedish longitudinal multi-cohort study
Source: J Gerontol A Biol Sci Med Sci. 2026 Sep 10;81(11):glag226. doi: 10.1093/gerona/glag226 (PMC13672823; doi:10.1093/gerona/glag226)
Supplement: Supplementary file 1 [file glag226_supplementary_data.docx]

**Supplementary materials**

**Disability trajectories in the last years of life: a Swedish longitudinal multi-cohort study**

**eTable 1.** Description of the included cohort studies

**eTable 2.** Sociodemographic characteristics of included and excluded participants

**eTable 3.** Model fit statistics and classification diagnostics for group-based trajectory models of trajectories of disability in relation to impending death among Swedish decedents who died at the age of 70 or older. Analyses are based on harmonized data from Swedish National E-infrastructure for Aging Research (NEAR) study. Study sample n = 2294

**eTable 4.** Estimated class proportions and parameter estimates from the three-class model of group-based trajectory analyses: trajectories of disability in relation to impending death among. Analyses are based on harmonized data from Swedish National E-infrastructure for Aging Research (NEAR) study. Study sample n = 2294

**eTable 5.** Participant characteristics across time-to-death (TTD) intervals and mean number of available ADL observations per participant.

**eTable 6.** Sociodemographic characteristics of those who provided data at one time point and those who provided data at more than two time points.

**eTable 7.** Model fit statistics and classification diagnostics for sensitivity analyses restricted to participants with ADL information at ≥3 time points (n = 699).

**eFigure 1** Study flowchart

**eFigure 2** Disability trajectories in relation to impending death among participants with ADL information available at more than two time points (sensitivity analysis, n = 699).

**eTable 1. Description of the included cohort studies**

| **Study** | **Baseline** | **Wave 2** | **Wave 3** | **Wave 4** | **Wave 5** |
| --- | --- | --- | --- | --- | --- |
| Betula | 1998-2000 | 2003-2005 | 2008-2011 | 2013-2014 | - |
| GENDER | 1999-2001 | 2003-2005 | - | - | - |
| OCTO-TWIN | 1997-2000 | 1999-2002 | - | - | - |
| SNAC-B | 2001-2003 | 2004-2006 | 2007-2009 | 2010-2012 | 2012-2015 |
| SNAC-K | 2001-2004 | 2004-2007 | 2007-2009 | 2010-2012 | 2013-2016 |
| SWEOLD | 2002 | 2004-2005 | 2010-2011 | - | - |

## **Description of the included cohort studies**

## Betula

The Betula Project is a longitudinal study on aging, memory, and dementia. Data was collected between 1988 and 2014. The study initially included 1,000 randomly selected individuals from Umeå Municipality across the age groups of 20, 35, 40, 45, 50, 55, 60, 65, 70, 75, and 80 years. Each age group consisted of 100 individuals, with a gender distribution similar to that of the general population—approximately equal numbers of men and women in the younger age groups, and about twice as many women as men among those aged 70 and older. The participants were followed every five years: in 1993, 1998, 2003, 2008, and 2013. At each assessment, participants underwent a comprehensive physical examination, including a blood test taken by a nurse, as well as a thorough memory assessment conducted by a memory examiner. Data was collected from approximately 4,700 participants. At each wave, data on around 2,000 variables per participant was gathered, covering demographics, health, disease, medication use, as well as social and cognitive factors. The Betula Project was approved by the Regional Ethics Review Board at Umeå University (Dnr: 2012-12-31M).

## GENDER

The GENDER study examines twin pairs born between 1906 and 1925. A survey on health and health behaviors was distributed in 1994, with responses from 1,210 twins from 605 pairs where both twins participated. The mean age at the start of the study was 74.43 years (SD 4.28), and all participants were Caucasian. Approval for the study was obtained from the Swedish Data Inspection Board and the Ethical Committee at Karolinska Institutet in Stockholm (Dnr 93:226), as well as from the Swedish Ethical Review Authority (Dnr: 2021–02095).

## OCTO-TWIN

The Origins of Variance in the Oldest-Old: Octogenarian Twins (OCTO-Twin) study aims to investigate the etiology of individual differences among twin pairs aged 80 and older across multiple domains, including health, functional capacity, cognitive function, psychological well-being, personality, and personal control. Twin pairs were selected from the Swedish Twin Registry. In the first wave, twins had to be born in 1913 or earlier, and both individuals in the pair had to agree to participate. At baseline (1991–94), 351 twin pairs (149 monozygotic and 202 same-sex dizygotic pairs) were examined (mean age: 83.6 years, 67% women). Follow-ups were conducted every two years with all surviving twins who agreed to participate. Data was collected over five waves spanning eight years. The study was approved by the Swedish Data Inspection Board and the Ethical Committee at Karolinska Institutet in Stockholm (Dnr 98–380).

## SNAC-B and SNAC-K

The Swedish National Study on Aging and Care (SNAC) is a longitudinal study that continuously collects data on cognitive functioning, genetics, socio-demographic factors, lifestyle, physical function, and chronic conditions through structured interviews and clinical examinations conducted by trained professionals. The purpose of SNAC is to increase our understanding of the aging process and to identify preventive strategies that can lead to improved health and care of older adults. SNAC includes four participating sites: SNAC-Blekinge (SNAC-B), SNAC-Kungsholmen (SNAC-K), SNAC-Nordanstig (SNAC-N), and SNAC-Skåne (SNAC-S). Baseline data collection was carried out between 2001 and 2004. In the current study we have included:

SNAC-B includes 1,817 participants. All phases of the project were approved by the Research Ethics Committee at Lund University on February 12, 2001 (Dnr LU 604-00) and the Swedish Ethical Review Authority (Dnr 2020-04378), Lund Division 2, Medicine.

SNAC-K includes 3363 participants. All phases of SNAC-K were approved by the Regional Ethical Review Board in Stockholm and the Swedish Ethical Review Authority (Dnrs: KI 01–114, 04–929/3, Ö26-2007, 2009/595–32, 2010/447–31/2, 2013/828–31/3, and 2016/730–31/1).

## SWEOLD

The Swedish Panel Study of Living Conditions of the Oldest Old (SWEOLD) is a nationally representative survey of the older population in Sweden. The sample consists of individuals who were previously part of the Swedish Level of Living Survey (LNU) and who have surpassed LNU’s age limit of 75 years. The first survey was conducted in 1992, followed by additional waves in 2002, 2004, 2010, and 2014. In the 2004 and 2014 waves, the survey included individuals aged 70 and older. SWEOLD data is linked to LNU, which has been conducted regularly since 1968. The number of participants ranges from 537 to 1,297. All phases of the project have been approved by the Swedish Ethical Review Authority (Dnrs: 2019–06324, 2020–04118, and 2021–00393).

**eTable 2. Sociodemographic characteristics of included and excluded participants**

|  | Excluded^a^  (*n* =605) | Included  (*n* = 2294) | *p* for difference |
| --- | --- | --- | --- |
| Women, % | 61.8 | 61.2 | 0.782 |
| Age at death, years, mean (SD) | 90.2 (6.8) | 89.8 (6.5) | 0.158 |
| Birth cohort |  |  | <0.001 |
| 1900–1918 | 29.8 | 41.7 |  |
| 1919–1929 | 55.7 | 46.1 |  |
| 1930–1944 | 14.5 | 12.3 |  |
| Education level, % |  |  | <0.001 |
| low | 68.0 | 56.6 |  |
| intermediate | 23.1 | 31.8 |  |
| high | 8.9 | 11.5 |  |

^a^ Comprises participants who had data on limitations in activities of daily living at only one time point during follow-up

**eTable 3. Model fit statistics and classification diagnostics for group-based trajectory models of trajectories of disability in relation to impending death among Swedish decedents who died at the age of 70 or older. Analyses are based on harmonized data from Swedish National E-infrastructure for Aging Research (NEAR) study. Study sample n = 2294**

| Number of latent classes | Polynomial order | AIC | BIC | Class proportions (%) | Entropy | Estimated posterior probabilities |
| --- | --- | --- | --- | --- | --- | --- |
| 1 | 2 | -6743.06 | -6751.67 |  |  |  |
| 2 | 2 2 | -5361.90 | -5381.90 | 68/32 | 0.78 | 0.95/0.94 |
| 3 | 2 2 2 | -5174.31 | -5205.86 | 58/38/9 | 0.68 | 0.87/0.84/0.88 |
| 4 | 2 2 2 2 | -5154.44 | -5197.48 | 61/26/7/6 | 0.60 | 0.80/0.75/0.64/0.84 |
| 5 | 2 2 2 2 2* | -5176.14 | -5230.66 | 52/1/7/31/10 | 0.45 | 0.52/0.61/0.49/0.81/.87 |
| **3** | **1 2 2** | **-5173.53** | **-5202.22** | **53/36/11** | **0.68** | **0.87/0.84/0.88** |

*Note.* The selected model is indicated in bold text *Model did not converge due to singularity

**eTable 4. Estimated class proportions and parameter estimates from the three-class model of group-based trajectory analyses: trajectories of disability in relation to impending death among. Analyses are based on harmonized data from Swedish National E-infrastructure for Aging Research (NEAR) study. Study sample n = 2294**

|  | **N** | **% of total** | **Intercept, mean (S.E.)** | **Linear term, mean (S.E.)** | **Quadratic term, mean (S.E.)** |
| --- | --- | --- | --- | --- | --- |
| Trajectory 1 | 1336 | 58 | -1.31 (0.12) | -0.81 (0.12) | NA |
| Trajectory 2 | 743 | 32 | 1.08 (0.04) | -0.34 (0.05) | -0.03 (0.01) |
| Trajectory 3 | 215 | 10 | 1.40 (0.05) | 0.05 (0.04) | -0.04 (0.01) |

*Note.* S.E. = standard error. NA = not applicable (the polynomial term was omitted for the trajectory in question)

**eTable 5.** **Participant characteristics across time-to-death (TTD) intervals and availability of longitudinal ADL data.**

|  | TTD 0-2 years  (*n* = 1110) | TTD 2-4 years  (*n* = 1287) | TTD 4-6 years  (*n* = 1321) | TTD 6-12 years  (*n* = 1601) | Mean number of available ADL observations per participant (SD) |
| --- | --- | --- | --- | --- | --- |
| Women, % | 62.0 | 60.4 | 63.4 | 60.1 | 2.3 (0.5) |
| Men, % | 38.0 | 39.6 | 36.6 | 39.9 | 2.3 (0.5) |
| Age at death, years, mean (SD) | 90.1 (6.2) | 90.1 (6.0) | 90.5 (6.2) | 89.6 (6.9) |  |
| Birth cohort |  |  |  |  |  |
| 1900–1918 | 48.0 | 44.4 | 43.5 | 32.8 | 2.3 (0.5) |
| 1919–1929 | 44.6 | 45.8 | 46.7 | 50.2 | 2.4 (0.5) |
| 1930–1944 | 7.4 | 9.8 | 9.8 | 17.1 | 2.2 (0.4) |
| Education level, % |  |  |  |  |  |
| low | 55.2 | 54.7 | 54.4 | 53.4 | 2.3 (0.5) |
| intermediate | 31.3 | 32.9 | 32.0 | 33.5 | 2.4 (0.5) |
| high | 10.6 | 12.4 | 11.3 | 13.1 | 2.2 (0.4) |

**eTable 6. Sociodemographic characteristics of those who provided data at one time point and those who provided data at more than two time points**

|  | ADL information from 2 timepoints  (*n* =1595) | ADL information from >2 timepoints  (*n* = 699) | *p* for difference |
| --- | --- | --- | --- |
| Women, % | 61.0 | 61.7 | 0.767 |
| Age at death, years, mean (SD) | 88.9 (6.8) | 91.7 (5.4) | <0.001 |
| Birth cohort |  |  | <0.001 |
| 1900–1918 | 42.7 | 39.3 |  |
| 1919–1929 | 42.6 | 53.9 |  |
| 1930–1944 | 14.7 | 6.7 |  |
| Education level*, % |  |  | <0.001 |
| low | 62.0 | 44.4 |  |
| intermediate | 28.4 | 37.9 |  |
| high | 9.6 | 15.9 |  |

*Education missing n=66

**eTable 7. Model fit statistics and classification diagnostics for sensitivity analyses restricted to participants with ADL information at ≥3 time points (n = 699).**

| Number of latent classes | Polynomial order | AIC | BIC | Class proportions (%) | Entropy | Estimated posterior probabilities |
| --- | --- | --- | --- | --- | --- | --- |
| 1 | 2 | -2818.66 | -2825.48 |  |  |  |
| 2 | 2 2 | -2206.41 | -2222.33 | 64/36 | 0.84 | 0.96/0.95 |
| 3 | 2 2 2 | -2097.14 | -2122.16 | 53/36/11 | 0.76 | 0.92/0.87/0.92 |
| 4 | 2 2 2 2* | -2090.96 | -2125.08 | 3/52/32/13 | 0.73 | 0.76/0.87/0.87/0.90 |
| 3 | 3 3 3 | -2123.90 | -2155.75 | 54/21/25 | 0.74 | 89/85/96 |
| **3** | **1 2 2** | **-2096.16** | **-2118.91** | **51/36/13** | **0.74** | **92/87/91** |

*Model did not converge due to singularity

**eFigure 1 Study flowchart**

**eFigure 2 Disability trajectories in relation to impending death among participants with ADL information available at more than two time points (sensitivity analysis, n = 699).**
